# Heat shock protein 90 inhibition in the endothelium

**DOI:** 10.3389/fmed.2023.1255488

**Published:** 2023-09-04

**Authors:** Nektarios Barabutis

**Affiliations:** School of Basic Pharmaceutical and Toxicological Sciences, College of Pharmacy, University of Louisiana Monroe, Monroe, LA, United States

**Keywords:** inflammation, lung injury, unfolded protein response, acute respiratory distress syndrome, COVID-19

## Introduction

Heat shock protein 90 (Hsp90) is a molecular chaperone assisting in the folding and maturation of a plethora of intracellular proteins, which participate in crucial functions and responses, including inflammation (1). Hsp90 inhibitors were developed—and are tested in clinical trials—to oppose cancers; and have been associated with anti-inflammatory activities (2). Those effects are not limited to malignant tissues, but are also applied in endothelial cells (3–5).

Endothelial inflammation promotes barrier dysfunction, tissue leak, lung edema, which are considered the hallmarks of acute respiratory distress syndrome (6). This is a respiratory disorder, associated with high mortality rates in the intensive care units, in septic patients. The COVID-19—related ARDS has caused more than 1,100,000 deaths in the Unites States (7), and efficient medicine to counteract it does not exist, so far.

Blocking the COVID-19—related cytokine storm it is a promising therapeutic strategy, and anti-inflammatory agents appear to improve patient survival. However, glucocorticoids are not efficient in cases of substantial inflammation, and monoclonal antibodies were developed to suppress the cytokine storm (8). IL-1 blockade delivered promising results (9). Indeed, there is an urgent need to develop new therapeutics against ARDS, utilizing robust anti-inflammatory agents.

Hsp90 inhibitors represent a promising therapeutic approach to oppose lung inflammatory disease, so to reinstate normal endothelial barrier function (10). In addition to their ability to block transcriptions factors which propel inflammatory responses (e.g., NFκB) (11), they can induce survival elements in charge of cell homeostasis, to ameliorate injury. P53 participates in those events.

P53 is a transcription factor which opposes the activities of NFkB in human tissues (12), and P53 deletion worsens LPS-induced injury in mice (13). P53 inhibition using pifithrin or small interfering RNA potentiated endothelial inflammation, while P53 augmentation exerted protective effects (14). The guardian of the genome mediates—at least in part—the effects of Hsp90 inhibition in the lungs, and mice overexpressing P53 were protected against inflammatory lung injury (15). Moreover, Hsp90 inhibition suppresses P53 phosphorylation, preventing P53 degradation (16, 17). The actin cytoskeleton is affected by P53, since this transcription factor can induce cortical actin, and suppress filamentous actin formation (18). The unfolded protein response (UPR) can also participate in the Hsp90 inhibitors—related effects in the endothelium.

UPR is a mechanism involved in cell fate, and can initiate repairing processes or induce cell death (19, 20). It is involved in endothelial barrier function. Recent studies suggest that UPR activation increases barrier integrity and reduces endothelial permeability, whereas its suppression is associated to impaired barrier function (21–25). Hsp90 inhibitors were shown to activate UPR sensors, as well as their downstream targets, in endothelial cells and mouse lungs (26, 27). The effects of Hsp90 inhibitors are also applied to brain microvascular cells, a component of the blood brain barrier. Specifically, those compounds protect brain cells against LPS (28) and oxidative stress (28, 29); in line with similar P53—mediated effects, *in vitro* (30, 31).

Hsp90 inhibitors may represent an exciting new possibility to counteract COVID-19. SARS-CoV-2 spike triggers barrier dysfunction and vascular leak via integrins and TGF-β signaling (32). The aforementioned compounds modulate SARS-CoV-2 spike protein subunit 1-induced human pulmonary microvascular endothelial activation and barrier dysfunction (33). They can also suppress SARS-CoV-2 assembly partially through induced M or N degradation (34). Interestingly, the oral Hsp90 inhibitor SNX-5422 attenuates SARS-CoV-2 replication and suppresses inflammation in airway cells (35).

## Discussion

Many questions are to be addressed about the specific mechanisms by which Hsp90 inhibition assists impaired/inflamed endothelial cells to survive, and affected tissues to recover. Which are the exact kinases mediating the effects of Hsp90 inhibitors toward P53 modulation, and how this molecular chaperone modulates UPR in endothelial cells? It was previous reported that IRE1α is involved in those phenomena, in cancers (36). Studies in genetically modified mice which do not express P53 and UPR sensors in their lung endothelium will most probably address those questions; to enrich our knowledge on the expanding Hsp90 universe.

## Author contributions

NB: Writing—original draft, Writing—review and editing.
